# Efficacy of *Euphorbia helioscopia* in context to a possible connection between antioxidant and antidiabetic activities: a comparative study of different extracts

**DOI:** 10.1186/s12906-021-03237-x

**Published:** 2021-02-12

**Authors:** Imtiaz Mustafa, Muhammad Naeem Faisal, Ghulam Hussain, Humaira Muzaffar, Muhammad Imran, Muhammad Umar Ijaz, Muhammad Umar Sohail, Arslan Iftikhar, Arslan Shaukat, Haseeb Anwar

**Affiliations:** 1 Department of Physiology, Faculty of Life Sciences, Government College University, Faisalabad, Pakistan; 2 Institute of Physiology and Pharmacology, University of Agriculture, Faisalabad, Pakistan; 3 Department of Food Science, Faculty of Life Sciences, Government College University, Faisalabad, Pakistan; 4 Department of Zoology, Wildlife and Fisheries, University of Agriculture, Faisalabad, Pakistan; 5 Biomedical Research Centre, Qatar University, Doha, Qatar

**Keywords:** *Euphorbia helioscopia*, Methanolic extract, Antioxidant, Antidiabetic

## Abstract

**Background:**

*Euphorbia helioscopia*, conventionally known as sun spurge, has been used as a traditional medicine to treat different diseases owing to its reported antitumor, antiviral and antioxidant activities.

**Methods:**

The current research was formulated to assess the in-vitro antioxidant and antidiabetic ability of *Euphorbia helioscopia* subsequent to the phytochemical analysis of its various extracts. For this purpose, methanol, ethanol and aqueous extracts were prepared using the whole dried plant. Phytochemical analysis of the extracts was done to evaluate the total flavonoid components (TFC) and total phenolic components (TPC) in the extracts. A total of seven phenolic and three flavonoid contents were documented and quantified using HPLC. Antioxidant values were found by DPPH^●^ assay, FRAP and ABTS assays. The antidiabetic potential of the extracts was evaluated by measuring the inhibition ability of the activity of enzymes α amylase and α glucosidase.

**Results:**

After analyzing statistically, the results showed that methanolic extract possesses the highest TFC and TPC values while aqueous extract encompassed the lowest level of these contents. *Invitro* results showed that methanolic extract of the *Euphorbia helioscopia* has the maximum antioxidant capability since it showed the highest scavenging ability towards the DPPH^●^ (IC_50_ value = 0.06 ± 0.02 mg/ml), FRAP (758.9 ± 25.1 μMFe^+ 2^/g), and ABTS (689 ± 25.94 μMTEq/g) due to the presence of high TPC (24.77 ± 0.35 mgGAEq/g) and TFC (17.95 ± 0.32 mgQEq/g) values. Antidiabetic activity in terms of inhibition potential of α amylase and α glucosidase activity was also observed maximum in methanolic extract having lowest IC_50_ value (0.4 ± 0.01 mg/ml and 0.45 ± 0.01 mg/ml respectively) and minimum in the aqueous extract (IC_50_ value = 0.57 ± 0.02 mg/ml and 0.76 ± 0.1 mg/ml respectively).

**Conclusion:**

The experiment outcomes have shown that *Euphorbia helioscopia* extracts used in the current study contain antioxidant and antidiabetic activities; however, it is highest in its methanolic extract. The presence of the same trend towards the highest antidiabetic activity of the methanolic extract in terms of maximum inhibiting activity of α amylase and α glucosidase enzymes suggests a close association of TFC and TPC in minimizing diabetes.

## Background

In recent decades, various natural plant extracts have exhibited significant antioxidant activity [1, 2]. These extracts encompass significant amounts of different bioactive molecules used in various pharmaceutical industry products [3, 4]. Particularly antioxidant molecules have upraised much attention because these secondary metabolites possess numerous pharmacological possessions [5]. One or more active ingredients from plants have been found in about 25% of all prescriptions [6]. Disproportionate reactive oxygen species that are derived from oxygen and nitrogen are the chief cause of the oxidative injury to tissues and organs [7, 8]. Oxidative damage has been reflected as a pathological process that playss a role to initiate and develop many diseases [9]. Oxidative stress can be induced by various factors including drugs, smoking, alcohol and environmental pollutants that may lead to hyperglycemia [10]. Various transcription factors that control the cellular responses to reactive oxygen species (ROS) can be triggered by ROS [11]. Increased ROS level is one of the important aspects in the progression of type 2 diabetes mellitus [12, 13]. In case of diabetes, ROS formation may be due to the oxidation of glucose, non-enzymatic glycation of proteins, and enhanced peroxidation of lipids that causes harm to the cells and enzymes leading to insulin resistance [14].

Euphorbiaceae family includes several medicinal plants across the world that contain a wide range of various therapeutic effects proposing the extent of chemical nature of extracts of plants of this group. *Euphorbia helioscopia* is a remarkable herbaceous annual medicinal plant of spurge family Euphorbiaceae indigenous to Asia, Europe and northern Africa. It contains almost 24 secondary metabolites, including euphornin, euphornins (B, C), euphoheliosnoid D, hemistepsin, helioscopinolide (B, C), licochalcone (A, B) echinatia, guaiane lactone, galabrone and 4′, 5,7-trihdroxyflavanone [15]. Because of the presence of a number of secondary metabolites, this plant has the diverse pharmacological effects including anti-inflammatory, vasodepressor activity, antimicrobial activity, antitumor, antioxidant and wound healing properties [16–20]. The plant has been used conventionally to cure different pathological conditions including skin diseases, warts, intestinal parasites, migraine and gonorrhea [21]. Leaves and stems of the plants are traditionally used as vermifuge and its seeds are used in cholera and constipation. Present research is designed to evaluate and compare the antioxidant and antidiabetic efficacy of methanolic, ethanolic and aqueous extracts of the *Euphorbia helioscopia.*

## Methods

### Procurement of plants

The plant *Euphorbia helioscopia* was locally collected from the fields of Ayub Agriculture Research Institute Faisalabad, Pakistan. The plant was identified by the expert Botanist with a voucher specimen numbered 247-bot-2020, and kept in the herbarium of the Department of Botany, Government College University Faisalabad, Pakistan.

### Extract preparation

After washing with distilled water, the plant was shade dried and grinded into a fine powder and 50 g of the powder was soaked for 72 h in 250 ml each of ethanol, methanol and distilled water with periodically stirring and mixing. The solutions were subsequently sieved through Whatman® filter paper. The extracts after filtration were evaporated and concentrated using a rotary evaporator (SCI100-Pro; SCILOGEX, USA) at 40 °C and transferred into labeled petri dishes and kept in incubator at 40 °C until dried properly. The percentage yield was calculated as 15.7, 11.2 and 13.9% for methanolic, ethanolic and aqueous extracts of the plant respectively. The extracts were stored at 4 °C till further analysis.

### Qualitative phytochemical analysis

Phytochemical analysis of methanolic, ethanolic, and water extracts of the plant was carried out qualitatively using standard methods as described by Singh and Bag [22] to verify the presence or absence of potentially active phytochemicals.

### Quantitative phytochemical estimation

#### Total phenolic constituents (mg of gallic acid equivalent/g dry weight of plant)

A volume of 30 μl plant extracts (1 mg/ml) was diluted with 30 μl of folin_ciocalteu reagent and 2.5% Na_2_CO_3_ (600 μl). After keeping at room temperature for 60 min, optical density was taken at 760 nm using a chemistry analyzer (Biolab-310). A gallic acid standard curve (0.789 to 200 μg/ml) was used for TPC evaluation [23].

#### Total flavonoid contents (mg of quercetin equivalent/g dry weight of plant)

Flavonoid contents were identified by using quercetin as a standard (0 to 100 μg/ml) following to the procedure previously adopted by Kumar et al. [23]. In a nutshell, each plant extract (100 μl) was mixed with distilled water (1 ml). After room temperature incubation for 5 min, AlCl_3_ (125 μl) and 5% NaNO_2_ (75 μl) were mixed and kept again at room temperature for 6 min. Then, 1 M NaOH (125 μl) and distilled water (2.5 ml) were added and absorbance was taken by using a chemistry analyzer (Biolab-310) at 540 nm.

#### Identification and quantification of phenolic constituents

A volume of 10 μl of plant extracts (0.1 g/ml in methanol) was injected in the HPLC system (HP 1050 gradient) with a detector (SPD-10AV) for estimation of phenolic profile. Stationary phase of Shim-Pack CLC-ODS C-18 column (5 μm,5 cm × 4.5 mm) Shimadzu, Japan® was used. A mixture of distilled water and glacial acetic acid in a v/v ratio of 24:0.4:320:56, was used as mobile phase. Different phenolic contents were measured with a 10 min linear gradient at room temperature [24].

### In-vitro antioxidant evaluation

#### FRAP assay (μmole Fe^2+^/g DW)

The FRAP was evaluated by using the procedure adopted previously by Dudonne et al. [25]. An amount of 3.995 ml of the final working solution [ten parts of 300 mM acetate buffer, one part of 10 mM 2,4,6-tri {2-pyridyl}-s-triazine in 40 mM HCL, one portion of 20 mM ferric Chloride] was diluted with 5 μl of the sample solution. The optical density was measured at 593 nm to evaluate the reducing ability. Results were determined by the comparison of absorbance with the standard curve constructed from different concentrations (0 to 1000 μMole) of ferrous sulphate (FeSO_4_) and expressed as μmole Fe^2+^/g dry weight of the plant.

#### ABTS assay (Trolox equivalent/g DW)

The ABTS scavenging potential was measured by using the ABTS assay previously used by Dudonne et al. [25]. A working solution of ABTS was made by mixing 7 mM of aqueous solution of ABTS and 2.5 mM of potassium persulfate in a 1:1 ratio. This ABTS working solution was further mixed with methanol to obtain an absorbance of almost 0.7 at wavelength 734 nm. After that, 5 μl of each plant extract solution was diluted with ABTS solution (3.995 ml). After keeping for 30 min at room temperature, optical density was taken at 734 nm and results were calculated by comparing the absorbances with a standard curve made from Trolox in various concentrations (0 to 800 μMole). The final values were shown as mg Trolox equiv./g of dry weight of the plant.

#### DPPH^●^ scavenging assay

Sample solution (5 μl) of different concentrations in methanol (5, 2.5, 1.25, 0.62 and 3.12 mg/ml) was mixed with 585 μl DPPH^●^ solution in methanol (0.2%) and kept at room temperature for almost twenty minutes. Then optical density was measured at 515 nm by using a chemistry analyzer (Biolab-310). The scavenging ability in percentage was measured by using the following formula:

$$ \mathrm{Scavenging}\ \left(\%\right)=100\times \frac{Abs^{\mathrm{bl}}-{Abs}^{\mathrm{sp}}}{Abs^{\mathrm{bl}}} $$

Where Abs^bl^ is the optical density of the DPPH^●^ blank solution and Abs^sp^ is the optical density of the extracts. A graph was plotted between percentage inhibition and extract concentration to calculate the concentration with 50% scavenging power (IC_50_) [26].

### Antidiabetic potential

#### Inhibition of α amylase activity

It was evaluated by the methodology as described previously [27] with a few modifications. Five serial diluted concentrations (0.312 to 5 mg/ml) of the plant extract (500 μl) and 500 μl of porcine pancreatic amylase solution (0.5 mgml^− 1^ in 0.02 M PBS with 6.9 pH having 0.006 M NaCl) was poured and incubated for 10 min at room temperature. Then 0.5 ml of 1% starch in 0.02 M PBS with 6.9 pH was mixed and kept at room temperature for 10 min and 1 ml of DNSA color reagent was added. The solution was then retained in a boiling water bath for 10 min to stop the reaction and diluted with 10 ml dH_2_O. At 540 nm, absorbance was measured by using a chemistry analyzer (Biolab-310®). Acarbose (Acr), a standard drug used to inhibit α amylase action, was also run in the same manner in different concentrations in place of the extracts. A blank solution was also run with 100% enzyme activity having no extract or the standard drug. The following formula was used to determine the percentage enzyme inhibition.

$$ \mathrm{Inhibition}\ \left(\%\right)=100\times \frac{\ \mathrm{abs}\ \mathrm{of}\ \mathrm{blank}-\mathrm{abs}\ \mathrm{of}\ \mathrm{sample}/\mathrm{standard}}{\mathrm{abs}\ \mathrm{of}\ \mathrm{blank}} $$

The concentration of the extract or the standard was calculated, having 50% inhibition of the enzyme activity (IC50) by constructing a graph of various quantities of the extracts and acarbose against their percent inhibition.

#### α glucosidase inhibitory activity

A volume of 980 μl of pNPG solution (290 mM β-D glucopyranoside in 20 mM citrate buffer with 5.6 pH) was mixed with 200 μl of five different concentrations of plant extracts and acarbose standard (5, 2.5, 1.25, 0.625, 0.312 mg/ml). This mixture after 5 min incubation at 37 °C was diluted with 20 μl of α glucosidase solution (1 U/ml) and kept at 35 °C for 40 min. The reaction was stopped by the addition of 200 μL of 6 N HCl, and optical density was measured at 405 nm by using chemistry analyzer (Biolab-310®). A blank solution was also run in a similar manner without the extract sample or acarbose.

$$ \mathrm{Percent}\ \mathrm{Inhibition}=100\times \frac{Abs^{\mathrm{bl}}-{Abs}^{\mathrm{sp}}}{Abs^{\mathrm{bl}}} $$

Where Abs^sp^ is the optical density of the sample and acarbose and Abs^bl^ is the optical density of blank. All the samples and acarbose were run in triplicate and IC_50_ value was calculated by constructing a chart of different quantities of the extracts and acarbose against their percent inhibition [27].

### Statistical study

All the measurements were calculated in triplicates and the data was analyzed for the mean ± standard deviation by using GraphPad Prism-8 software. Correlations were calculated by means of bivariate linear correlations (*p* < 0.05 and *p* < 0.01), using Pearson’s correlation coefficient (r) in Microsoft office Excel 2010.

## Results

### Qualitative phytochemical analysis

Qualitative analysis of all three extracts of the plant is expressed in the Table. 1. It indicates the presence of different phytochemicals including alkaloids, phenols, anthraquinones, flavonoids, reducing sugar, saponins, terpenoids, steroids and tannins in MthEh, EthEh and AqEh.

**Table 1:** Qualitative Analysis of *Euphorbia helioscopia*

No	Phytochemicals	MthEh	EthEh	AqEh
1	Alkaloids	+ +	+ + +	+ +
2	Phenols	+ + +	+ +	+
3	Anthraquinones	–	–	–
4	Flavonoids	+ + +	+ +	+
5	Reducing sugar	+	+ +	–
6	Saponins	+	+	–
7	Terpenoids	–	+	+
8	Steroids	+	+	–
9	Tannins	+ +	+ +	+

(+++); Strongly positive, (++); moderately positive, (+); weakly positive, (−): not detected

*MthEh* Methanolic extract of *Euphorbia helioscopia*, *EthEh* Ethanolic extract of *Euphorbia helioscopia*, *AqEh* Aqueous extract of *Euphorbia helioscopia*

### Total flavonoid and phenolic contents

**Fig. 1:**
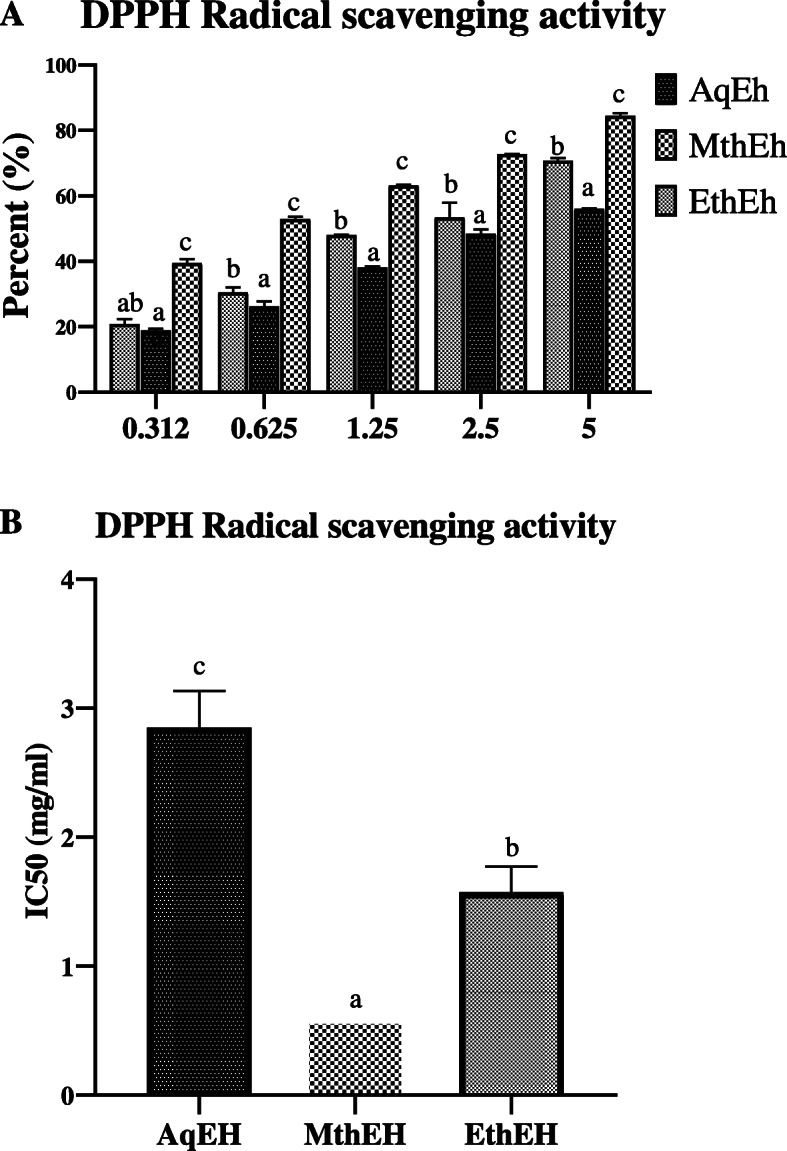
**a** 2,2-diphenyl-1-picrylhydrazyl (DPPH^●^) radical scavenging activity of five different concentrations of different extracts of *Euphorbia helioscopia.* **b** IC_50_ value of DPPH^●^ radical scavenging activity of different extracts of *Euphorbia helioscopia.* Results are Mean ± Standard Deviation of three replicates of each group. Different lower case letters (^a^ to ^c^) above the bars indicate a significant difference between groups (*P* ≤ 0.05). MthEh: methanolic extract of *Euphorbia helioscopia;* EthEh: Ethanolic extract of *Euphorbia helioscopia;* AqEh: Aqueous extract of *Euphorbia helioscopia*

Results indicated that both the TFC and TPC were significantly higher in MthEh (17.95 ± 0.32 mgQE/g and 24.77 ± 0.35 mgGAE/g respectively) in comparison to EthEh (11.27 ± 0.38 mgQE/g and 13.58 ± 0.43 mgGAE/g respectively) and AqEh (3.25 ± 0.95 mgQE/g and 4.63 ± 0.69 mgGAE/g respectively). Figure 1a & b exhibited that TPC and TFC in the *Euphorbia helioscopia* extracts were in the following sequence: MthEh > EthEh > AqEh.

### Identification and quantification of phenolic contents

Overall, seven phenolic contents, including gallic acid, chlorogenic acid, hydroxy benzoic acid, caffeic acid, P-cumaric acid, vanillic acid, ferulic acid, and three flavonoid contents comprising catechin acid, quercetin and rutin were identified and quantified by HPLC. Phenolic acids found in ethanolic extract included gallic acid, chlorogenic acid, caffeic acid, P-cumaric acid and ferulic acid, while phenolic compounds found in methanolic extract included gallic acid, hydroxybenzoic acid, chlorogenic acid, caffeic acid and vanillic acid and those found in aqueous extract included gallic acid, hydroxybenzoic acid, chlorogenic acid and caffeic acid as presented in Table. 2. Fig. 4a, b & c are showing the chromatograms of different extracts of *Euphorbia helioscopia* plant. Among the phenolic contents, chlorogenic acid was found maximum in MtEh (2368.06.41 ± 81.84 mg/g) and AqEh (1072.95 ± 41.25 mg/g) while gallic acid was found maximum in EthEh (829.41 ± 52.31 mg/g). Three flavonoid contents were quantified in all three extracts (catechin acid, quercetin and rutin), among which rutin was found in maximum quantity in all the three extracts with an order as MthEh>EthEh>AqEh (Table. 2).

**Table 2:** HPLC Analysis of Different Extracts of *Euphorbia helioscopia* for Total Phenolic and Flavonoid Contents

Compound Name	MthEh (mg/g)	EthEh (mg/g)	AqEh (mg/g)
Phenolic Contents			
Gallic Acid	608.62 ± 49.23 ^b^	829.41 ± 52.31 ^a^	297.21 ± 21.87 ^c^
HydroxyBenzoic Acid	N.D	29.98 ± 0.91 ^a^	9.43 ± 0.43 ^b^
Chlorogenic acid	2368.06 ± 81.84 ^a^	765.64 ± 21.02 ^c^	1072.95 ± 41.25 ^b^
Caffic acid	42.69 ± 1.21 ^a^	13.53 ± 0.82 ^b^	13.53 ± 0.61 ^b^
Vanlic acid	N.D	17.40 ± 0.51	N.D
P-cumaric acid	133.15 ± 7.05	N.D	N.D
Ferulic acid	2.04 ± 0.27	N.D	N.D
Flavonoid Contents			
Catechin acid	814.87 ± 102.7 ^a^	774.7 ± 62.31 ^b^	386.33 ± 24.44 ^c^
Quercetin	531.94 ± 31.39 ^a^	226.94 ± 9.81 ^b^	92.1 ± 7.91 ^c^
Rutin	2149.39 ± 119.28^a^	1774.60 ± 94.83 ^b^	741.11 ± 37.21 ^c^

Results are expressed as Means±SD (standard deviation). Values that do not share a superscript letter (a to c) in the same row are significantly different (*p* ≤ 0.5)

*MthEh* Methanolic extract of *Euphorbia helioscopia*, *EthEh* Ethanolic extract of *Euphorbia helioscopia*, *AqEh* Aqueous extract of *Euphorbia helioscopia*, *N.D* Not detected

### In vitro antioxidant evaluation

#### FRAP assay (FeSo4 (μmoleFe^2+^/g DW)) and ABTS assay (μMTrolox Equiv./g DW)

Results of both FRAP and ABTS assay are expressed in Table. 3 that indicated that the MthEh possess the highest reducing potential of Fe^3+^ into Fe^2+^ (758.9 μmoleFe^2+^/g) as compared to the EthEh (457.85 μmoleFe^2+^/g) and AqEh (303.49 μmoleFe^2+^/g) presented in Table. 3. The same trend was seen in terms of scavenging ABTS radical being maximum in MthEh (689 μMTE/g) followed by EthEh (575.17 μMTE/g) and AqEh (287.39 μMTE/g) shown in Table. 3.

**Table 3:** Total phenolic content, total flavonoid content, Ferric reducing antioxidant potential (FRAP), and Trolox equivalent antioxidant capacity (TEAC; ABTS Assay) of different extracts of *Euphorbia helioscopia*

	MthEh	EthEh	AqEh
TPC (mg GAEq/g)	24.77^c^ ± 0.35	13.58^b^ ± 0.43	4.63 ^a^ ± 0.69
TFC (mgQEq/g)	17.95^c^ ± 0.32	11.27 ^b^ ± 0.38	3.25 ^a^ ± 0.93
FRAP (μMFe^+ 2^/g)	758.90^c^ ± 25.21	457.85 ^b^ ± 13.15	303.49 ^a^ ± 4.45
TEAC (μMTEq/g)	689.00^c^ ± 25.94	575.17 ^b^ ± 7.52	287.39 ^a^ ± 13.90

Results are expressed as Means±SD (standard deviation). Values that do not share a superscript letter (a to c) in the same row are significantly different (*p* ≤ 0.5)

*MthEh* Methanolic extract of *Euphorbia helioscopia*, *EthEh* Ethanolic extract of *Euphorbia helioscopia*, *AqEh* Aqueous extract of *Euphorbia helioscopia*

#### DPPH^●^ radical scavenging activity

The results showed a concentration-dependent increase in DPPH^●^ scavenging activity in all three extracts with maximum activity in the MthEh (Fig. 1a) with the lowest IC_50_ value (0.6 ± 0.02 mg/ml) in comparison of EthEh (1.6 ± 0.2 mg/ml) and AqEh (2.8 ± 0.3 mg/ml) as presented in Fig. 1b.

### Antidiabetic potential

#### Inhibition of α amylase activity

**Fig. 2:**
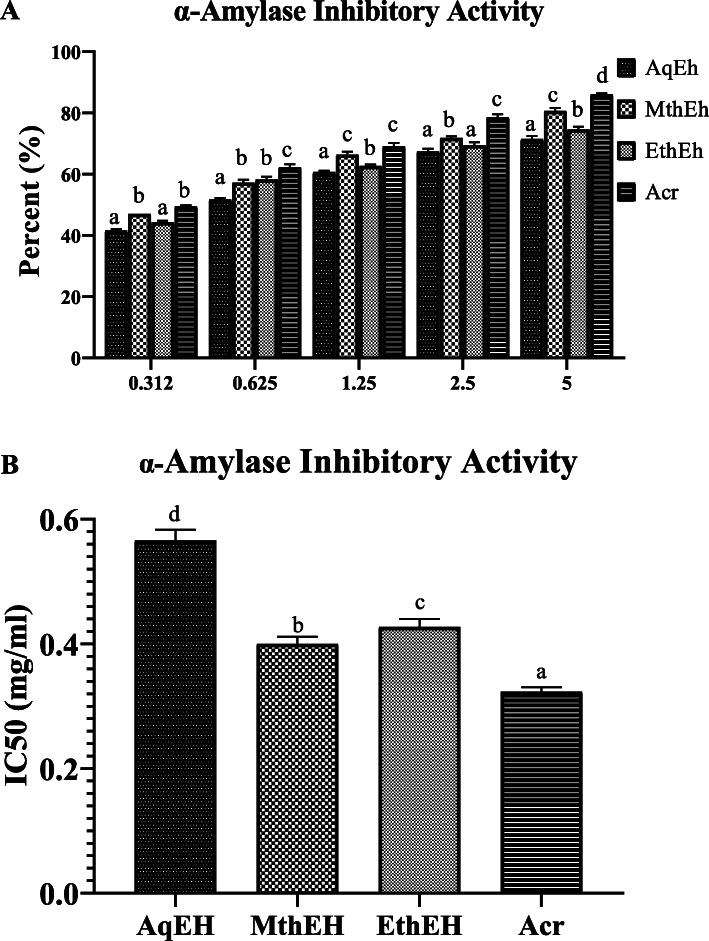
**a** α Amylase inhibitory activity of five different concentrations of different extracts of *Euphorbia helioscopia.* **b** IC_50_ value of α Amylase inhibitory activity of different extracts of *Euphorbia helioscopia.* Results are Mean ± Standard Deviation of three replicates of each group. Different lowercase letters (^a^ to ^d^) above the bars indicate a significant difference between groups (*P* ≤ 0.05). MthEh: methanolic extract of *Euphorbia helioscopia;* EthEh: Ethanolic extract of *Euphorbia helioscopia;* AqEh: Aqueous extract of *Euphorbia helioscopia*; Acr: Acarbose (standard drug)

The result showed that *Euphorbia helioscopia* extracts contained the appreciable amylase inhibition activity in a concentration-dependent way (Fig. 2a). Among all three extracts, MthEh showed the maximum αamylase inhibitory activity in terms of having the lowest IC_50_ value of 0.4 ± 0.01 mg/ml^,^ which was slightly higher than the standard drug acarbose (0.32 ± 0.008 mg/ml) and lower than that of AqEh (0.57 ± 0.02 mg/ml) and EthEh (0.43 ± 0.01 mg/ml) (Fig. 2b).

#### Inhibition of α Glucosidase activity

**Fig. 3:**
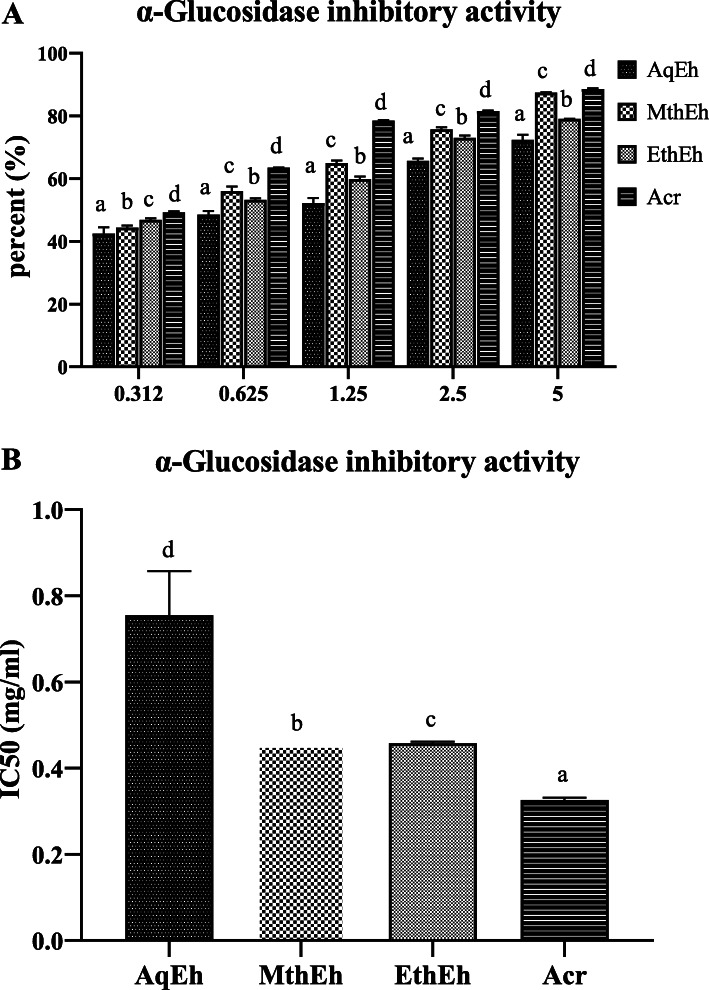
**a** α glucosidase inhibitory activity of five different concentrations of different extracts of *Euphorbia helioscopia.* **b** IC_50_ value of α Glucosidase inhibitory activity of different extracts of *Euphorbia helioscopia.* Results are Mean ± Standard Deviation of three replicates of each group. Different lowercase letters (^a^ to ^d^) above the bars indicate a significant difference between groups (*P* ≤ 0.05). MthEh: methanolic extract of *Euphorbia helioscopia;* EthEh: Ethanolic extract of *Euphorbia helioscopia;* AqEh: Aqueous extract of *Euphorbia helioscopia*; Acr: Acarbose (standard drug)

The potential of the extracts and acarbose to inhibit α glucosidase enzyme activity was also seen in a concentration-dependent increase (Fig. 3a). The aqueous extract showed the highest IC_50_ value, which means that it has the lowest α glucosidase inhibitory activity in increasing order as AqEh<EthEh<MthEh<Acr (Fig. 3b).

#### Correlation among phytochemicals (TFC and TPC) and antioxidant potential

Table. 4 indicates a positive correlation of about 99% among TPC and FRAP (r = 0.993) and about 91% positive correlation among TPC and ABTS scavenging action (r = 0.953). Moreover, a strong negative correlation of about 98% was seen among TPC and IC_50_ concentration of DPPH^●^ radical scavenging action and TPC (r = − 0.989), which indicates that the increase in TPC will minimize the quantity of the extract required to scavenge 50% of the DPPH^●^. Correlation between TFC and antioxidant activity was also in the same manner (Table. 4).

**Table 4:** Pearson’s correlation coefficients of phytochemicals in E. H with different antioxidant and antidiabetic parameters

	Phytochemicals		Antioxidant activity			Antidiabetic activity	
	TPC	TFC	FRAP	TEAC	IC_**50**_ value of DPPH	IC_**50**_ value of α-amylase inhibitory activity	IC_**50**_ value of α-glucosidase inhibitory activity
**Phytochemicals**							
**TPC**	1		0.993**	0.953**	−0.989**	−0.999**	−0.848**
**TFC**	**–**	1	0.972**	0.982**	−0.9995**	− 0.997**	− 0.904**
**Antioxidant activity**							
**FRAP**	0.993**	0.972**	1	**–**	**–**	−0.98**	− 0.778**
**TEAC**	0.953**	0.982**	**–**	1	**–**	−0.992**	−0.969**
**IC**_**50**_ **value of DPPH**	−0.989**	−0.9995**	**–**	**–**	1	0.996**	0.917**
**Antidiabetic activity**							
**IC**_**50**_ **value of α-amylase inhibitory activity**	−0.999**	−0.997**	− 0.98**	−0.992	0.996**	1	**–**
**IC**_**50**_ **value of α-glucosidase inhibitory activity**	−0.848**	−0.904**	− 0.778**	−0.969**	0.917**	**–**	1

** Correlation is significant at (*p* ≤ 0.01)

#### Correlation of phytochemicals (TFC and TPC) and antioxidants with antidiabetic activity

A strong negative correlation was seen among TPC and IC_50_ values of α amylase inhibitory activity (r = − 0.998). The correlation of TFC with IC_50_ of α amylase enzyme inhibition ability (r = − 0.999) also suggests an increase in TFC will increase the α amylase inhibitory activity (Table. 4). Correlation coefficients for the correlation of IC_50_ values of α amylase enzyme inhibition activity with IC_50_ of DPPH^●^ radical scavenging activity also show strong positive correlation r values are given in the Table. 4. A strong negative correlation of about 98% was seen among FRAP and IC_50_ of α amylase enzyme inhibition action. Moreover, the correlation among ABTS and IC_50_ of α amylase enzyme inhibition ability was found almost 99% (r = 0.9916). Whereas r value for the IC_50_ values of DPPH^●^ with α amylase inhibitory activity was found 0.9996.

The correlation of TFC and TPC with IC_50_ ofα glucosidase enzyme inhibition action was found as r = − 0.904 and r = − 0.848, respectively. A similar correlation was seen among FRAP and IC_50_ value of α glucosidase inhibitory activity (r = − 0.778), whereas ABTS and IC_50_ of α glucosidase enzyme inhibition activity was found negatively correlated with each other as r = − 0.969. The correlation of the IC_50_ value of DPPH^●^ radical scavenging activity with IC_50_ value of alpha glucosidase inhibitory activities was seen positive (r = 0.917) (Table. 4).

## Discussion

Medicinal plants possess huge quantities of antioxidant agents that play a significant role in the adsorption and neutralization of free radicals. These phytochemicals produce noteworthy antioxidant capacities in the plants that ultimately play a pivotal role in curing several human diseases [4]. Outcomes of the current study have shown that MthEh possessed the highest phenolic contents 24.77 ± 0.35 mgGAE/gDW as compared to the EthEh 13.58 ± 0.43 mgGAE/gDW and AqEh 4.63 ± 0.69 mgGAE/gDW extract. Similarly, the TFC were seen in high concentrations in MthEh 17.95 ± 0.32 mgQE/g in contrast to the EthEh 11.27 ± 0.38 mgQE/g and AqEh 3.25 ± 0.95 mgQE/g. A previous study by Nepote et al. [28] also suggested that methanol solvent is ideal for the extraction of various phenolic components. In another study by Ben Mohamed Maoulainine et al. [29] revealed that methanolic extract of the *Euphorbia helioscopia* possesses a high concentration of TPC and TFC as compared to the TPC and TFC in ethanolic extract.

**Fig. 4:**
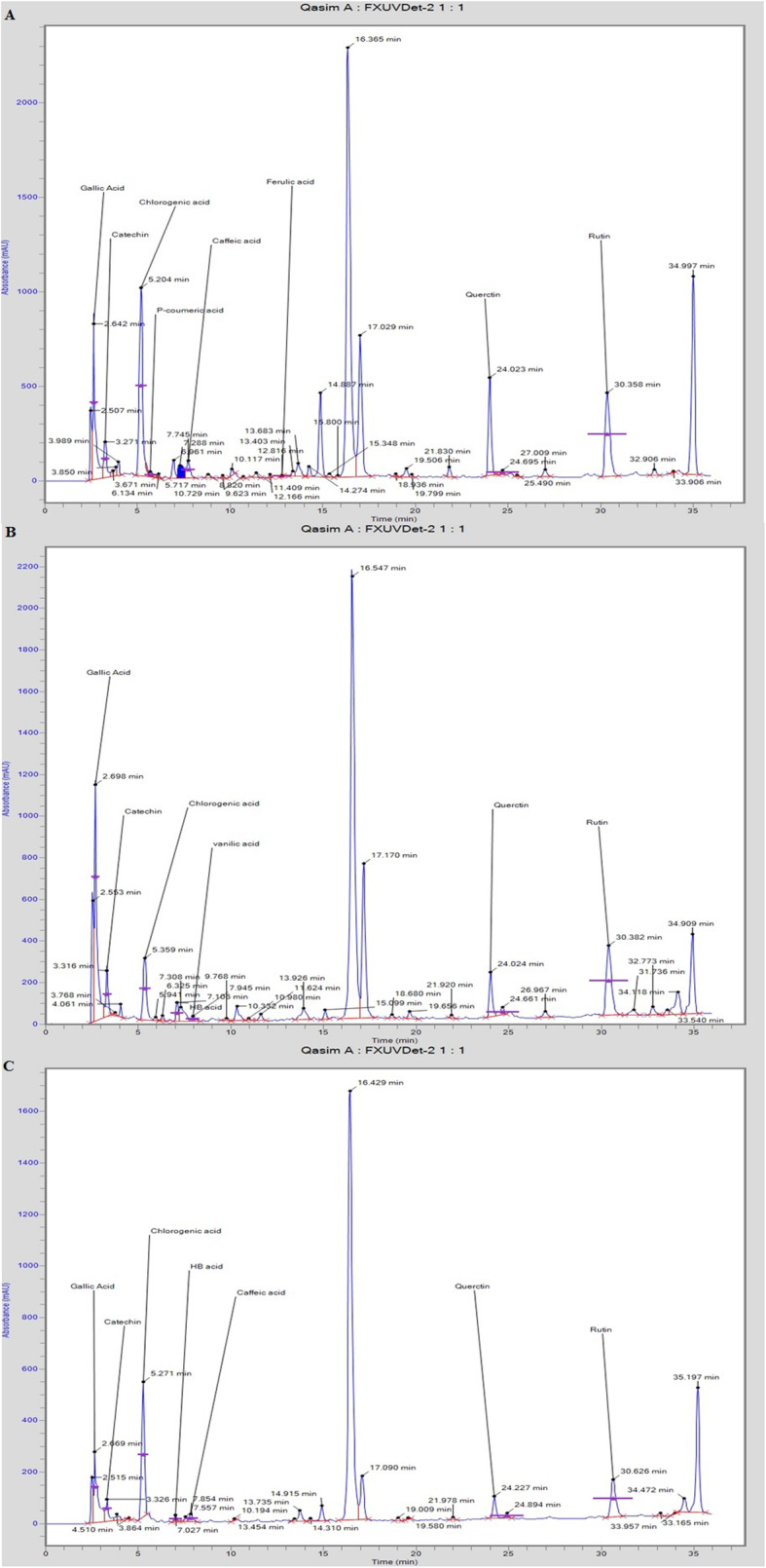
HPLC chromatograms of different extracts of *Euphorbia helioscopia.* **a** HPLC Chromatogram of methanolic extract of *Euphorbia helioscopia*. **b** HPLC Chromatogram of ethanolic extract of *Euphorbia helioscopia*. **c** HPLC Chromatogram of aqueous extract of *Euphorbia helioscopia*

Since it is speculated that calorimetric assays may not be able to give a complete picture of the quality and quantity of different flavonoid and phenolic components in any extract [30], we underwent HPLC technique to validate the existence of phenolic and flavonoid components in the extracts (Table 2; Fig. 4). It was observed that three phenolic acids gallic acid, caffeic acid, and chlorogenic acid, and all the three flavonoid constituents were commonly found in all the three extracts. Among the phenolic contents, chlorogenic acid was found maximum in MtEh and AqEh, while gallic acid was found maximum in EthEh. Several pieces of research have demonstrated that phenolic compounds possess effective antioxidant power and radical scavenging potential [31]. The antioxidant power of phenolic contents is primarily because of their redox activities, due to which they play a role as reducing mediators, proton donors, and oxygen quencher [32]. In the present study, DPPH^●^, FRAP and ABTS assays were used to evaluate the antioxidant potential of the plant extracts. Data showed a significant antioxidant effect of all plant extracts. FRAP results showed that the MthEh possessed the highest antioxidant ability in comparison with EthEh and AqEh. Likewise, the ABTS result also verified that of MthEh contained the highest antioxidant ability among all extracts. The free radicals scavenging ability of all extracts was also assessed through DPPH^●^ in-vitro assay that substantiated the previous findings. Various studies confirm the close association of phenolic and flavonoids with antioxidant activity [33].

The current study also suggested a close relationship of total flavonoid and phenolic components with the DPPH^●^, FRAP, and ABTS results. Table. 4 shows a 99% correlation among FRAP and TPC (r = 0.993) and a95% correlation among ABTS results and TPC (r = 0.953). This study also shows that the increased DPPH^●^ scavenging ability of MthEh is also due to the increased TPC and TFC of MthEh. That’s why a strong negative correlation of 98% was seen among TPC and IC_50_ concentration of DPPH^●^ radical scavenging activity, suggesting that an increase in the TPC will increase the DPPH^●^ scavenging ability (r = − 0.989). A similar relation of TFC was also seen with DPPH^●^ scavenging ability, FRAP and ABTS radical scavenging ability of different extract of *Euphorbia helioscopia* (Table. 4). Phenolic acids and flavonoid contents, in general, contribute towards an important class of bioactive constituents, which play a key role as antioxidants [34], and act by neutralizing the hydroxyl ions [35], superoxide anion radicals [36], and lipid proxy radicals [37]. A previous study by Chandra et al. [38] described that total phenolic content contributes about 61 and 75% to the antioxidant properties in the tower garden and field-grown crops, respectively. They also described the correlation among the total flavonoids content and antioxidant activity that flavonoids contribute 32 and 30% in the tower garden and field grown crops. The methanol extract of the leaves of *Euphorbia helioscopia* can increase the antioxidant enzymes, including catalase, superoxide dismutase, and glutathione [39]. The study of Ben Mohamed Maoulainine et al. [29] also explained that the methanolic extract that showed higher TPC and TFC values showed maximum DPPH^●^ radical scavenging activity in terms of IC_50_ value as compared to the ethanolic extract, which justifies the results of our study.

By virtue of antioxidant and anti-inflammatory properties, phenolic contents in plants prevent the oxidation process and protect cell injury to avoid the danger of degenerative diseases, including diabetes mellitus type II [40–42]. The α amylase, produced from the salivary glands and pancreas, plays a main role in the digestion of carbohydrates by breaking the alpha bonds of polysaccharides. Likewise, α glucosidase is another significant enzyme present in the intestinal lumen and membrane brush border, help to digest the carbohydrates by converting starch and oligosaccharide into monosaccharides [43]. Thus, both these enzymes work to increase postprandial blood glucose level, which is strongly associated with micro and macrovascular complications in diabetes mellitus. Inhibitors of these enzymes are anticipated to suppress these enzymes’ activity, delaying starch conversion into disaccharides and monosaccharides, which would ultimately decrease the glucose absorption and drop the postprandial glucose levels [44]. Drugs like acarbose and miglitol are competitive inhibitors of α glucosidases and α amylase that work to delay carbohydrates’ digestion. These synthetic drugs may still result in diarrhea, softening of feces, and abdominal discomfort [45]. Through in-vitro analysis of α amylase and α glucosidase inhibitory activity, we aimed to evaluate the natural inhibitors of these enzymes present in different extracts of *Euphorbia helioscopia*. Our results showed that MthEh possessed the maximum inhibitory ability of α amylase enzyme activity, having the lowest IC_50_ compared to EthEh and AqEh. Similarly, the results indicated that MthEh possessed the maximum α glucosidase inhibitory activity and the lowest IC_50_ value among all extracts. Many researches described the antidiabetic activities of the plants belong to Euphorbiaceae family such as *Euphorbia hirta* was tested for its antidiabetic potential in streptozotocin induced diabetic mice and the results showed that the plant inhibited the activity of α amylase and significantly reduced blood glucose level in hyperglycemic mice [46]. Tuhin et al. [47] evaluated *Euphorbia hirta* wound healing property in the diabetic rats and the plant also lowered the blood glucose level.

The correlation of TFC and TPC with the antidiabetic potential of the plant extracts has shown that greater the TFC and TPC, greater will be the ability of the extracts to inhibit α amylase and α glucosidase enzyme activities (Table. 4). The study also indicated that the plant’s aqueous extract possessed the lowest concentration of flavonoids with the maximum α amylase and α glucosidase inhibitory activity. The mechanisms of action that play a role in the inhibition of these enzymes by the plant ingredients are not known properly. Still, a few studies suggest that flavonoids might induce some conformational changes in these enzymes’ structures, hence blocking their activity. The findings of earlier research by Narkhede et al. [44] presented that gallic acid may inhibit the α amylase enzyme, which coincides with our results. However, α amylase and α glucosidase inhibitory activity of our study show contrary results in which MthEh showed more antidiabetic activity than EthEh. This difference might be due to other plant ingredients like tannins, which also play an important role in inhibiting α amylase activity [44].

## Conclusions

In conclusion, our study revealed that methanolic extract of the *Euphorbia helioscopia* has the highest antioxidant capability among other extracts since it contains the highest FRAP and scavenging ability towards the radicals ABTS and DPPH^●,^ of high TFC and TPC values. In terms of α amylase and α glucosidase inhibition, methanolic extract shows maximum antidiabetic activity. These extracts must be further analyzed and characterized for future research to identify and synthesize antidiabetic drugs by searching the mode of action of different constituents towards the management of diabetes.

## Data Availability

The datasets used and/or analyzed during the current study are available from the corresponding author on reasonable request.

## Abbreviations

FRAP: Ferric Reducing Antioxidant Potential
ABTS: (2,2′-azino-bis (3-ethylbenzothiazoline-6-sulfonic acid))
DPPH^●^: (1,1-Diphenyl-2-picrylhydrazyl)
MthEh: methanolic extract of *Euphorbia helioscopia*
EthEh: Ethanolic extract of *Euphorbia helioscopia*
AqEh: Aqueous extract of *Euphorbia helioscopia*
DW: Dry Weight
Acr: Acarbose
TFC: Total flavonoid contents
TPC: Total phenolic contents
